# Crystal structure of the acyclic form of 1-de­oxy-1-[(4-methoxyphenyl)(methyl)amino]-d-fructose

**DOI:** 10.1107/S2056989018000099

**Published:** 2018-01-09

**Authors:** Valeri V. Mossine, Charles L. Barnes, Thomas P. Mawhinney

**Affiliations:** a Department of Biochemistry, University of Missouri, Columbia, MO 65211, USA; b Department of Chemistry, University of Missouri, Columbia, MO 65211, USA

**Keywords:** crystal structure, d-fructosamine, acyclic carbohydrate, Hirshfeld surface analysis

## Abstract

The title compound, C_14_H_21_NO_6_, (I), crystallizes exclusively in the acyclic *keto* form. In solution of (I), the acyclic tautomer represents only 10% of the population in equilibrium, with the other 90% consisting of β-pyran­ose, β-furan­ose, α-pyran­ose, and α-furan­ose cyclic forms. The carbohydrate chain in (I) has a zigzag conformation and the aromatic amine group has a transitional *sp* ^2^/*sp* ^3^ geometry. Bond lengths and valence angles in the carbohydrate portion compare well with the average values for related acyclic polyol structures. All of the hydroxyl groups are involved in inter­molecular hydrogen bonding and form a two-dimensional network of infinite chains, which are inter­linked by intra­molecular hydrogen bonds and organized into *R*_8_^8^(16) homodromic ring patterns. A comparative Hirshfeld surfaces analysis of (I) and four other 1-amino-1-de­oxy-d-fructose derivatives suggests the balance of hydro­philic/hydro­phobic inter­actions plays a role in the crystal packing, favoring the acyclic isomer.

## Abstract 2

The monoclinic unit contains a rare acyclic *keto* tautomer of the amino sugar involved in the extensive hydrogen-bonding patterns. The acyclic conformation is a minor species in the compound’s solution.

## Chemical context   

Reducing carbohydrates, for instance aldoses (glucose, mannose, xylose) or ketoses (fructose, ribulose), mutarotate in solutions such that the predominant species in equilibrium consist of cyclic pyran­ose and furan­ose hemiacetals or hemiketals, respectively (Angyal, 1992 ▸). Free aldehyde or ketone forms are thermodynamically unfavorable and normally comprise less than 1% of the population in the equilibria. Crystallization of reducing monosaccharides naturally affords the most populous, predominantly pyran­ose, anomers (Jeffrey, 1990 ▸). Previously, we have demonstrated that exceptions to this rule may be found among 1-amino-1-de­oxy-ketoses. Only four acyclic ketosamine structures have been accurately characterized by X-ray diffraction so far (Mossine *et al.*, 1995 ▸, 2002 ▸, 2009 ▸); however, it was suggested that the hydro­phobic nature of the amino substituents may play a supporting role in stabilization of the unique structures (Mossine *et al.*, 2009 ▸). Given that the concept of acyclic inter­mediates is essential for understanding the mechanisms of many enzymatic and non-enzymatic transformations of carbohydrates in general (see, for example, Buchholz & Seibel, 2008 ▸; Wang *et al.*, 2014 ▸) and natural fructosamines in particular (Nursten, 2005 ▸), the availability of precise structural knowledge on the open-chain 1-amino-1-de­oxy-ketoses is of inter­est to the field. We report here on the structure of title compound, alternatively named as d-fructose-*N*-methyl-*p*-anisidine, C_14_H_21_NO_6_, (I), aiming to expand this knowledge.
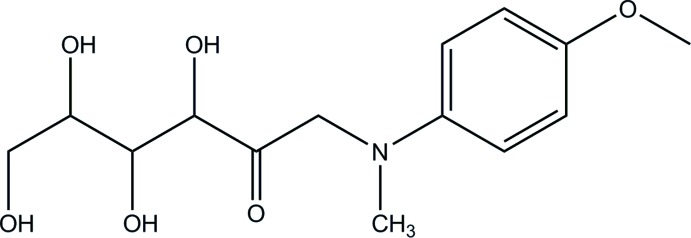


## Structural commentary   

**Figure 1:**
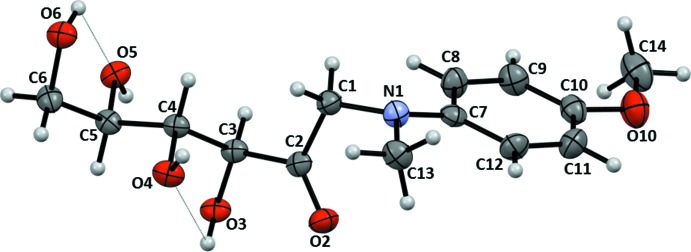
Atomic numbering and displacement ellipsoids at the 50% probability level for (I). Intra­molecular O—H⋯O inter­actions are shown as dotted lines.

**Figure 2:**
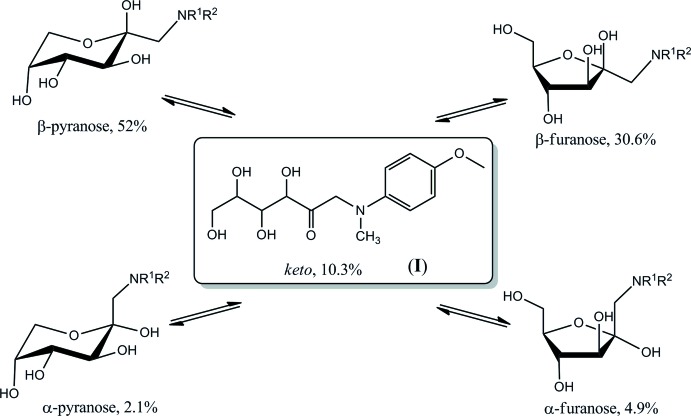
Isomerization equilibrium of (I) in solution.

The mol­ecular structure and atomic numbering for (I) are shown in Fig. 1 ▸. The mol­ecule may be considered as a conjugate of a carbohydrate, 1-amino-1-de­oxy-d-fructose, and an aromatic amine, *N*-methyl-*p*-anisidine, which are joined through the common amino group. The carbohydrate portion in (I) exists in the acyclic *keto* form. Remarkably, in the aqueous solution of (I), the acyclic *keto* form is a minor constituent of the established equilibrium, at 10.3% of the total population as follows from the ^13^C NMR data (Table 1 ▸). The predominant β-pyran­ose anomer (52.0%) and smaller proportions of the β-furan­ose, α-pyran­ose, and α-furan­ose cyclic forms constitute the rest of the equilibrium composition (Fig. 2 ▸).

**Figure 3:**
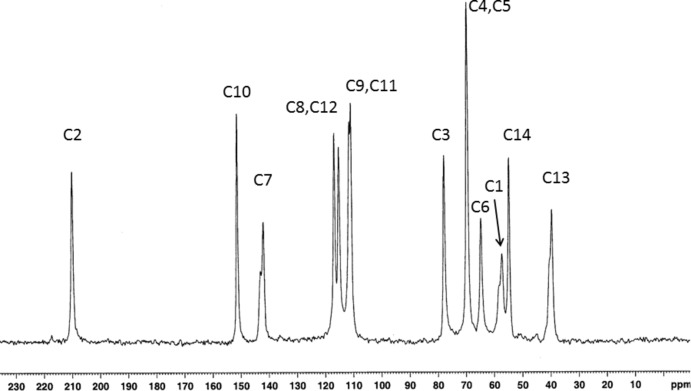
Solid-state ^13^C NMR spectrum of powdered crystalline (I).

The carbohydrate fragment of the mol­ecule is in the *zigzag* conformation, having four out of six of its carbon atoms, C3, C4, C5, and C6, located in one plane. The conformation around the carbonyl group is also nearly flat and involves atoms N1, C1, C2, O2, C3, and O3, with the carbonyl O2 in the *syn-periplanar* position with respect to both N1 and O3 [respective torsion angles are 11.7 (3) and −7.0 (3)°]. This type of conformation is preferred for the β-amino­carbonyl group, due to influence of the σ_C—H_ → σ_C=O_* and σ_C—H_ → π_C=O_* hyperconjugation in conditions when inter­action between the nitro­gen lone pair (LP) and the carbonyl π*-system is not significant (Ducati *et al.*, 2006 ▸). Indeed, the LP—N1—C1—C2 torsion angle estimate is close to 180° in (I). In the sugar portion of (I), the average C—O bond distances (1.43 ± 0.01Å) and the valence angles in hydroxyl groups are close to the average values for a number of crystalline alditol structures (Jeffrey & Kim, 1970 ▸) and acyclic ketosamines (Mossine *et al.*, 1995 ▸, 2002 ▸, 2009 ▸). Two heteroatom contacts, O3—H⋯O4 and O6—H⋯O5, although weakly directional (Table 2 ▸), are cooperatively integrated into the hydrogen-bonding scheme (see Section 3) and thus are good candidates to qualify for intra­molecular hydrogen bonds. The tertiary amino group geometry is a flattened pyramid, with the distance from the N1 apex to the C1–C7–C13 base being 0.219 Å and the average base-face dihedral angle 17.2°. The N1—C7 distance, at 1.403 Å, is significantly shorter than the distances from N1 to the aliphatic carbons C1 and C13 [1.444 (3) and 1.455 (3) Å]. Such geometry is characteristic for amino groups with a mixed *sp* ^3^/*sp* ^2^ hybridization, likely due to a partial resonance of the nitro­gen *p*-electrons with a neighboring π-system, such as the benzene ring in (I). In the solid-state ^13^C NMR spectrum (Fig. 3 ▸), the peaks corresponding to the carbons C1, C7, and C13 are split, indicating a conformational dimorphism of the tertiary amino group, possibly due to an inversion of configuration at the N1 atom.

## Supra­molecular features   

**Figure 4:**
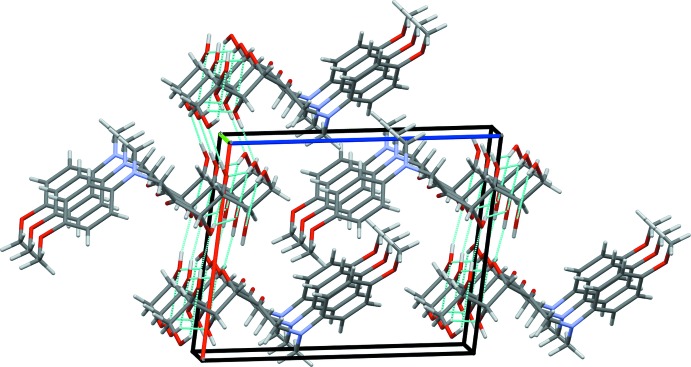
The mol­ecular packing in (I). Color code for crystallographic axes: red – *a*, green – *b*, blue – *c*. Hydrogen bonds are shown as cyan dotted lines.

**Figure 5:**
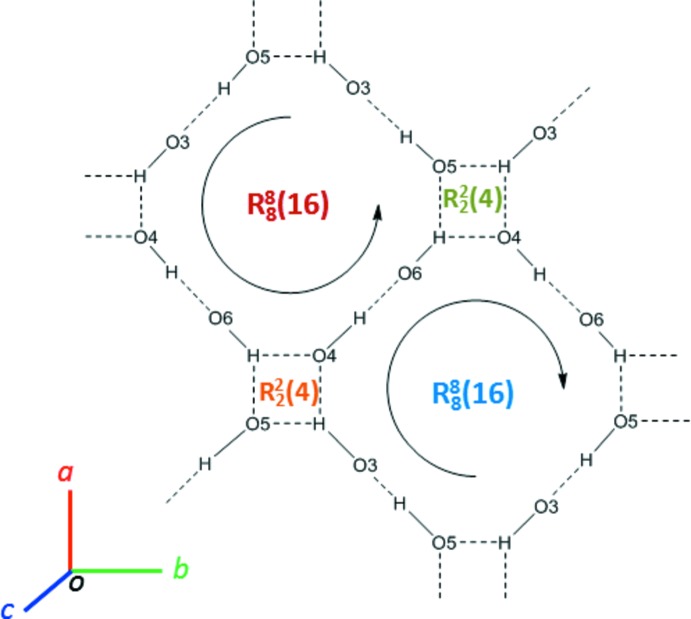
Hydrogen-bonding pattern in the crystal structure of (I).

**Figure 6:**
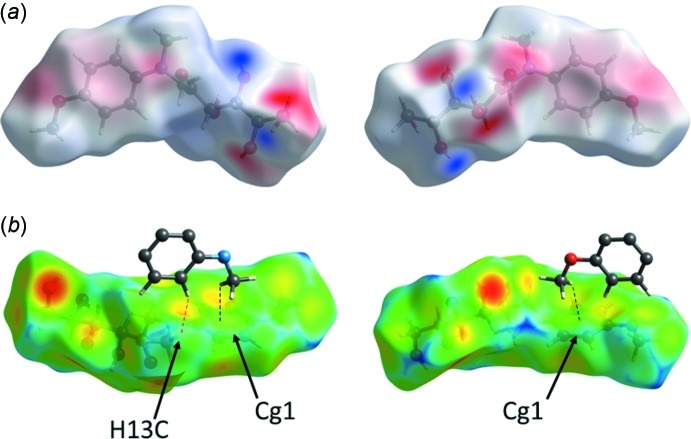
Views of the Hirshfeld surface for (I) mapped over: (*a*) the electrostatic potential in the range −0.0966 to +0.1843 a.u. The red and blue colors represent the distribution of the negative and positive electrostatic potential, respectively; (*b*) the *d*_e_ function, in the range 0.683 to 2.484 Å, calculated for the external contact atoms in the crystal. Shown are mol­ecular fragments involved in the C—H⋯π inter­actions (black dotted lines) and the shortest H⋯H contact (red dotted line).

**Figure 7:**
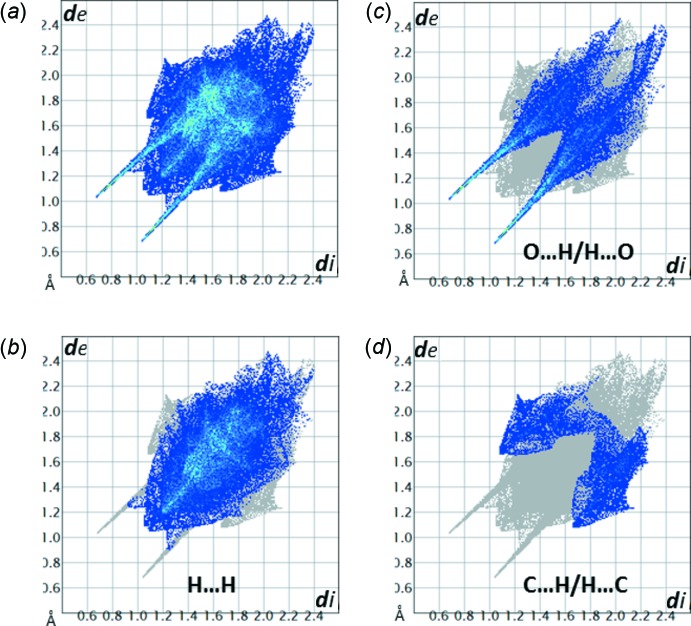
(*a*) The full two-dimensional fingerprint plot for (I) and those delineated for the specific contacts: (*b*) O⋯H; (*c*) H⋯H; (*d*) C⋯H.

Compound (I) crystallizes in the monoclinic space group *P*2_1_, with two equivalent mol­ecules per unit cell. The mol­ecular packing of (I) features ‘hydro­philic’ and ‘hydro­phobic’ layers propagating in the *ab* plane (Fig. 4 ▸). The carbohydrate residues form a two-dimensional network of hydrogen bonds organized as a system of two homodromic infinite chains, with ⋯O3—H⋯O5—H⋯ and ⋯O4—H⋯O6—H⋯ recurrent sequences of inter­molecular hydrogen bonds. These chains are topologically connected by the intra­molecular short heteroatom contacts O3—H⋯O4 and O6—H⋯O5. Basic hydrogen-bonding patterns of the resulting network are depicted in Fig. 5 ▸ and include fused homodromic 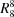
(16) and anti­dromic 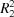
(4) rings (the pattern notation according to Bernstein *et al.*, 1995 ▸). The inter­molecular heteroatom contacts that define the hydrogen bonding in (I) are not confined exclusively to the carbohydrate portion of the mol­ecule (Fig. 6 ▸), however. In addition, there are two close C—H⋯O2 contacts involving the carbonyl group, and two short C—H⋯π contacts between the methyl groups and the benzene ring centroids (*Cg*1), which may qualify as weak hydrogen bonds (Table 3 ▸, Fig. 6 ▸). The Hirshfeld surface analysis (Spackman & Jayatilaka, 2009 ▸) revealed that a major proportion of the inter­molecular contacts in crystal structure of (I) is provided by non- or low-polar inter­actions of the H⋯H and C⋯H type (Fig. 7 ▸ and Table 4 ▸).

## Database survey   

A search of SciFinder, Google Scholar, and the Cambridge Structural Database (Groom *et al.*, 2016 ▸) by both structure and chemical names for 1-de­oxy-1-(*N*-methyl-*p*-meth­oxy­phenyl­amino)-d-fructose returned no references; hence compound (I) is new. There are four closely related structures, namely d-fructose-*N*-methyl-*p*-toluidine (FruNMpti, CCDC 717802), d-fructose-*p*-toluidine (Frupti, CCDC 126260), d-fructose-*N*-ethyl-*p*-chloro­aniline (FruNEpca, CCDC 717803), and d-fructose-*N*-allyl­aniline (FruNAlla, CCDC 717417). Each of these 1-amino-1-de­oxy-d-fructose derivatives features an aromatic substituent at the amino group. They also display a similar to (I) distribution of the cyclic and acyclic tautomeric forms in solutions (Table 1 ▸). However, only FruNMpti and FruNEpca were reported to adopt the acyclic *keto* conformations in crystalline state. Frupti (Gomez de Anderez *et al.*, 1996 ▸), FruNAlla (Mossine *et al.*, 2009*a* ▸), as well as the rest of the 1-amino-1-de­oxy-d-fructose derivatives whose structures were solved by X-ray diffraction methods (about 15 structures so far), crystallize in the β-d-fructo­pyran­ose anomeric form (Table 1 ▸). The unusual propensity of some 1-amino-1-de­oxy-d-fructose derivatives, including (I), to crystallize in a thermo­dynamically unfavored acyclic form is difficult to explain, given that the number of the available solved structures is thus far too small. Modelling the energies of inter­molecular inter­actions experienced by these mol­ecules in solutions *versus* crystal environments was beyond the goals of the current study. However, some initial clues can be derived from analysis of data compiled in Table 1 ▸, this work, as well as in Table 1 ▸ from our previous study (Mossine *et al.*, 2009 ▸). First, only fructosamine derivatives decorated with an aromatic amino substituent can, but not always, crystallize as the acyclic *keto* tautomer. As pointed out in Section 2, a neighboring π-system may resonate with the amine *p*-electrons thus making them unavailable for σ-bonding. Next, among *N*-aryl derivatives of 1-amino-1-de­oxy-d-fructose, only those lacking a proton bound to the tertiary amino group can crystallize in the acyclic form. Indeed, no hydrogen bonds involving N1 were detected in acyclic (I), FruNMpti or FruNEpca (Mossine *et al.*, 2009 ▸). In contrast, the structures of Frupti, FruAlla and all the rest of the 1-amino-1-de­oxy-d-fructose derivatives reveal at least one hydrogen-mediated intra­molecular heteroatom contact between the amino nitro­gen atom and an oxygen atom originating from the carbohydrate portion of the mol­ecule, most often the anomeric O2. Thus, the inability of the amino group to form stable intra­molecular hydrogen bonds with the carbohydrate portion plays a role in stabilization of the acyclic tautomer. Finally, a comparative Hirshfeld surfaces analysis (Table 4 ▸) of these structures suggests that the extended linear conformation of the acyclic tautomer may require more of the ‘hydro­philic space’ available in the crystal structure, as compared to the pyran­ose anomers. This argument also seems to be supported by an observation that an increase in size of the *N*-substituents, such as from methyl or ethyl (in FruNMpti and FruNEpca) to allyl or butyl (in FruAlla and d-fructose-*N*-butyl­aniline), leads to a loss of propensity to crystallize in the acyclic form (Mossine *et al.*, 2009 ▸).

## Synthesis and crystallization   

The preparation of (I) was performed following a protocol described previously (Mossine *et al.*, 2009 ▸). Briefly, a mixture of 5.4 g (0.03 moles) of d-glucose, 2.7 g (0.022 moles) of *p*-anisidine and 0.55 mL of 3-mercaptopropionic acid catalyst/anti­oxidant was stirred for 6 h in 12 mL of iso­propanol in a screw-capped glass vial at 360 K. The reaction progress was followed by TLC. The purification step included an ion-exchange on Amberlite IRN-77 (H^+^), with 0.2 *M* NH_4_OH in 50% ethanol as an eluant, and was followed by flash filtration on a short silica column using 5% MeOH in CH_2_Cl_2_ as an eluant. Crystallization of the compound was aided by the addition of a small amount of acetone to the syrupy evaporation residue. The crystals were filtered off, washed with acetone and dried *in vacuo* over CaCl_2_, yield 1.6 g (27%, based on starting amine) of colorless prisms. Major (β-pyran­­ose anomer) peaks (ppm) in the ^13^C NMR spectrum in D_2_O/pyridine: 154.14 (C10); 148.12 (C7); 117.86, 117.11 (C8, C12); 116.96, 116.80 (C9, C11); 101.56 (C2); 72.98 (C4); 71.99 (C3, C5); 65.90 (C6); 63.21 (C1); 57.84 (C14); 42.62 (C13). The ^13^C CPMAS–TOSS spectrum of finely powdered crystalline (I) is shown in Fig. 3 ▸ and the minor peak assignments are listed in Supplementary Table S1.

## Refinement   

Crystal data, data collection and structure refinement details are summarized in Table 5 ▸. Hy­droxy and nitro­gen-bound H atoms were located in difference-Fourier analyses and were allowed to refine fully. Other H atoms were placed at calculated positions and treated as riding, with C—H = 0.98 Å (meth­yl), 0.99 Å (methyl­ene) or 1.00 Å (methine) and with *U*_iso_(H) = 1.2*U*_eq_(methine or methyl­ene) or 1.5*U*_eq_(meth­yl). The Flack absolute structure parameter determined [0.3 (5) for 1149 quotients (Parsons *et al.*, 2013 ▸)] is consistent with the (3*S*,4*R*,5*R*) configuration which was assigned for this chain system on the basis of the known configuration for the starting material d-glucose (McNaught, 1996 ▸).

## Supplementary Material

Crystal structure: contains datablock(s) I. DOI: 10.1107/S2056989018000099/qm2121sup1.cif

Structure factors: contains datablock(s) I. DOI: 10.1107/S2056989018000099/qm2121Isup2.hkl

CCDC reference: 1811885

Additional supporting information:  crystallographic information; 3D view; checkCIF report

## Figures and Tables

**Table 1:** Distribution (%) of cyclic and acyclic forms of some 1-amino-1-de­oxy-D-fructose derivatives in D_2_O/pyridine (1:1) at 293 K, as estimated from the ^13^C NMR spectra, and in the crystalline state

Compound	α-pyran­ose	β-pyran­ose	α-furan­ose	β-furan­ose	acyclic, *keto*	Crystalline isomers
(I)	2.1	52.0	4.9	30.6	10.3	acyclic *keto*
FruNMpti^*a*^	2.1	49.9	4.8	32.2	11.0	acyclic *keto*
FruNEpca^*a*^	2.0	48.7	4.2	32.3	12.7	acyclic *keto*
Frupti^*a*,*b*^	3.5	61.0	9.4	24.2	1.9	β-pyran­ose
FruAlla^*c*^	2.2	47.4	4.5	33.6	12.3	β-pyran­ose
Fructosamine^*d*^	5.0	70.8	11.2	12.3	0.8	β-pyran­ose
FruAib^*e*^	3.0	75.6	10.1	10.4	<0.7	β-pyran­ose

Notes: (*a*) Mossine *et al.* (2009 ▸); (*b*) Gomez de Anderez *et al.* (1996 ▸); (*c*) Mossine *et al.* (2009*a* ▸); (*d*) Mossine *et al.* (2009*b* ▸); (*e*) Mossine *et al.* (2018 ▸).

**Table 2:** Hydrogen-bond geometry (Å, °)

*D*—H⋯*A*	*D*—H	H⋯*A*	*D*⋯*A*	*D*—H⋯*A*
O3—H3*O*⋯O4	0.87 (3)	2.67 (3)	2.962 (2)	101 (3)
O3—H3*O*⋯O5^i^	0.87 (3)	1.94 (3)	2.747 (2)	153 (2)
O4—H4*O*⋯O6^ii^	0.80 (3)	1.90 (3)	2.700 (2)	176 (3)
O5—H5*O*⋯O3^iii^	0.86 (3)	1.86 (3)	2.702 (2)	165 (3)
O6—H6*O*⋯O5	0.82 (3)	2.56 (3)	2.918 (2)	108 (3)
O6—H6*O*⋯O4^iv^	0.82 (3)	1.95 (3)	2.704 (2)	154 (3)

Symmetry codes: (i) 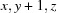; (ii) 
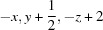
; (iii) 
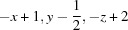
; (iv) 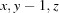.

**Table 3:** Suspected C—H⋯*A* contacts (Å, °)

C—H⋯*A*	C—H	H⋯*A*	C⋯*A*	C—H⋯*A*	Symmetry
C1—H1*A*⋯O2	0.99	2.48	3.386 (3)	152	*x*, *y* − 1, *z*
C14—H14*B*⋯O2	0.98	2.52	3.311 (3)	138	-*x* + 1, *y* − 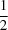 , −*z* + 1
C14—H14*A*⋯*Cg*1	0.98	2.95	3.747 (3)	139	-*x* + 1, *y* − 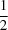 , −*z* + 1
C13—H13*C*⋯*Cg*1	0.98	2.80	3.539 (2)	133	-*x*, *y* + 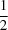 , −*z* + 1

**Table 4:** Contributions (%) of specific contact types to the Hirshfeld surfaces of 1-amino-1-de­oxy-D-fructose derivatives

Compound	Conformation	O⋯H	H⋯H	C⋯H	Other contacts
(I)	acyclic *keto*	32.3	52.8	13.2	N⋯H 1.6; C⋯C 0.1
FruNMpti^*a*^	acyclic *keto*	26.5	59.8	11.8	N⋯H 1.6; C⋯C 0.3
FruNEpca^*a*^	acyclic *keto*	23.1	50.1	8.6	N⋯C 0.5; C⋯C 1.3; Cl⋯H 13.1; Cl⋯C 3.4
FruNAlla^*b*^	β-pyran­ose	15.2	67.7	16.9	C⋯C 0.1
FruNBn_2_^*c*^	β-pyran­ose	16.5	64.2	19.2	C⋯O 0.1
TagNMBn^*d*^	α-pyran­ose	20.6	65.8	13.5	O⋯O 0.1

Notes: (*a*) Mossine *et al.* (2009 ▸); (*b*) Mossine *et al.* (2009*a* ▸); (*c*) Hou *et al.* (2001 ▸); (*d*) Pérez *et al.* (1978 ▸).

**Table 5:** Experimental details

Crystal data	
Chemical formula	C_14_H_21_NO_6_
*M*_r_	299.32
Crystal system, space group	Monoclinic, *P*2_1_
Temperature (K)	100
*a*, *b*, *c* (Å)	10.8002 (15), 5.1439 (7), 13.3931 (19)
β (°)	98.382 (1)
*V* (Å^3^)	736.11 (18)
*Z*	2
Radiation type	Mo *K*α
μ (mm^−1^)	0.11
Crystal size (mm)	0.35 × 0.15 × 0.12

Data collection	
Diffractometer	Bruker APEXII CCD area detector
Absorption correction	Multi-scan (*SADABS*; Sheldrick (2003 ▸)
*T*_min_, *T*_max_	0.88, 0.99
No. of measured, independent and observed [*I* > 2σ(*I*)] reflections	8031, 2993, 2788
*R*_int_	0.026
(sin θ/λ)_max_ (Å^−1^)	0.625

Refinement	
*R*[*F* ^2^ > 2σ(*F* ^2^)], *wR*(*F* ^2^), *S*	0.031, 0.082, 1.04
No. of reflections	2993
No. of parameters	208
No. of restraints	1
H-atom treatment	H atoms treated by a mixture of independent and constrained refinement
Δρ_max_, Δρ_min_ (e Å^−3^)	0.19, −0.15
Absolute structure	Flack *x* determined using 1149 quotients [(*I* ^+^)−(*I* ^−^)]/[(*I* ^+^)+(*I* ^−^)] (Parsons *et al.*, 2013 ▸)
Absolute structure parameter	0.3 (5)

Computer programs: *SMART* and *SAINT* (Bruker, 1998 ▸), *SHELXS97* (Sheldrick, 2008 ▸), *SHELXL2017/1* (Sheldrick, 2015 ▸), *X-SEED* (Barbour, 2001 ▸), *Mercury* (Macrae *et al.*, 2008 ▸), *CIFTAB* (Sheldrick, 2008 ▸) and *publCIF* (Westrip, 2010 ▸).

## supplementary crystallographic information

### Crystal data

**Table:**

C_14_H_21_NO_6_	*F*(000) = 320
*M**_r_* = 299.32	*D*_x_ = 1.350 Mg m^−^^3^
Monoclinic, *P*2_1_	Mo *K*α radiation, λ = 0.71073 Å
*a* = 10.8002 (15) Å	Cell parameters from 3774 reflections
*b* = 5.1439 (7) Å	θ = 2.6–25.8°
*c* = 13.3931 (19) Å	µ = 0.11 mm^−^^1^
β = 98.382 (1)°	*T* = 100 K
*V* = 736.11 (18) Å^3^	Prism, colourless
*Z* = 2	0.35 × 0.15 × 0.12 mm

### Data collection

**Table:**

Bruker APEXII CCD area detector diffractometer	2788 reflections with *I* > 2σ(*I*)
ω scans	*R*_int_ = 0.026
Absorption correction: multi-scan (SADABS; Sheldrick (2003)	θ_max_ = 26.4°, θ_min_ = 1.9°
*T*_min_ = 0.88, *T*_max_ = 0.99	*h* = −13→13
8031 measured reflections	*k* = −6→6
2993 independent reflections	*l* = −16→16

### Refinement

**Table:**

Refinement on *F*^2^	Hydrogen site location: mixed
Least-squares matrix: full	H atoms treated by a mixture of independent and constrained refinement
*R*[*F*^2^ > 2σ(*F*^2^)] = 0.031	*w* = 1/[σ^2^(*F*_o_^2^) + (0.0443*P*)^2^ + 0.0803*P*] where *P* = (*F*_o_^2^ + 2*F*_c_^2^)/3
*wR*(*F*^2^) = 0.082	(Δ/σ)_max_ < 0.001
*S* = 1.04	Δρ_max_ = 0.19 e Å^−^^3^
2993 reflections	Δρ_min_ = −0.15 e Å^−^^3^
208 parameters	Absolute structure: Flack *x* determined using 1149 quotients [(*I*^+^)-(*I*^-^)]/[(*I*^+^)+(*I*^-^)] (Parsons *et al*., 2013)
1 restraint	Absolute structure parameter: 0.3 (5)

### Special details

**Table:**

Geometry. All esds (except the esd in the dihedral angle between two l.s. planes) are estimated using the full covariance matrix. The cell esds are taken into account individually in the estimation of esds in distances, angles and torsion angles; correlations between esds in cell parameters are only used when they are defined by crystal symmetry. An approximate (isotropic) treatment of cell esds is used for estimating esds involving l.s. planes.

### Fractional atomic coordinates and isotropic or equivalent isotropic displacement parameters (Å^2^)

**Table:**

	*x*	*y*	*z*	*U*_iso_*/*U*_eq_	
N1	0.10655 (16)	0.5973 (4)	0.64477 (12)	0.0275 (4)	
C1	0.15040 (19)	0.4862 (4)	0.74257 (15)	0.0267 (4)	
H1A	0.189953	0.316322	0.732685	0.032*	
H1B	0.077318	0.453299	0.777617	0.032*	
O2	0.26415 (14)	0.8787 (3)	0.79127 (12)	0.0321 (4)	
C2	0.24391 (18)	0.6543 (4)	0.81040 (15)	0.0240 (4)	
O3	0.40317 (13)	0.6914 (3)	0.95778 (11)	0.0268 (3)	
C3	0.31015 (18)	0.5253 (4)	0.90646 (15)	0.0229 (4)	
H3	0.352434	0.364549	0.886440	0.027*	
O4	0.13795 (14)	0.6653 (3)	0.99012 (12)	0.0281 (3)	
C4	0.21360 (18)	0.4440 (4)	0.97432 (14)	0.0220 (4)	
H4	0.159267	0.302656	0.940569	0.026*	
O5	0.35348 (13)	0.1285 (3)	1.06292 (11)	0.0270 (3)	
C5	0.27762 (17)	0.3493 (4)	1.07730 (15)	0.0231 (4)	
H5	0.332604	0.491086	1.109907	0.028*	
O6	0.09673 (13)	0.0803 (3)	1.10474 (11)	0.0285 (3)	
C6	0.18494 (19)	0.2727 (5)	1.14715 (15)	0.0285 (5)	
H6A	0.231641	0.204885	1.210924	0.034*	
H6B	0.138815	0.429609	1.163615	0.034*	
C7	0.18576 (18)	0.5977 (4)	0.57050 (15)	0.0250 (4)	
C8	0.2825 (2)	0.4181 (5)	0.57062 (16)	0.0322 (5)	
H8	0.300894	0.301939	0.625936	0.039*	
C9	0.3529 (2)	0.4053 (5)	0.49151 (17)	0.0352 (5)	
H9	0.417860	0.280262	0.493123	0.042*	
O10	0.39239 (16)	0.5762 (4)	0.32846 (12)	0.0437 (4)	
C10	0.3286 (2)	0.5736 (5)	0.41076 (16)	0.0319 (5)	
C11	0.2348 (2)	0.7560 (5)	0.41043 (17)	0.0352 (5)	
H11	0.218288	0.874400	0.355652	0.042*	
C12	0.1644 (2)	0.7690 (5)	0.48871 (16)	0.0316 (5)	
H12	0.100474	0.896471	0.486890	0.038*	
C13	0.0158 (2)	0.8065 (5)	0.64424 (17)	0.0330 (5)	
H13A	0.059076	0.974339	0.646201	0.050*	
H13B	−0.027328	0.790523	0.703438	0.050*	
H13C	−0.045390	0.795655	0.582714	0.050*	
C14	0.4858 (2)	0.3812 (7)	0.3254 (2)	0.0474 (7)	
H14A	0.551236	0.401318	0.383834	0.071*	
H14B	0.522812	0.399739	0.263176	0.071*	
H14C	0.447653	0.208769	0.327161	0.071*	
H5O	0.430 (3)	0.175 (7)	1.061 (2)	0.050 (8)*	
H4O	0.069 (3)	0.643 (7)	0.959 (2)	0.052 (9)*	
H6O	0.133 (3)	−0.034 (6)	1.078 (2)	0.047 (8)*	
H3O	0.368 (2)	0.835 (6)	0.974 (2)	0.041 (8)*	

### Atomic displacement parameters (Å^2^)

**Table:**

	*U*^11^	*U*^22^	*U*^33^	*U*^12^	*U*^13^	*U*^23^
N1	0.0270 (9)	0.0286 (9)	0.0267 (9)	0.0036 (8)	0.0032 (7)	0.0003 (7)
C1	0.0277 (11)	0.0254 (10)	0.0268 (10)	−0.0020 (9)	0.0032 (8)	0.0011 (8)
O2	0.0344 (8)	0.0240 (8)	0.0372 (9)	−0.0023 (7)	0.0026 (7)	0.0027 (7)
C2	0.0207 (9)	0.0242 (10)	0.0288 (10)	0.0003 (8)	0.0099 (8)	−0.0007 (8)
O3	0.0166 (7)	0.0272 (8)	0.0365 (8)	−0.0027 (6)	0.0038 (6)	−0.0025 (6)
C3	0.0180 (9)	0.0223 (10)	0.0287 (10)	−0.0003 (7)	0.0048 (8)	−0.0013 (8)
O4	0.0178 (7)	0.0281 (8)	0.0383 (8)	0.0029 (6)	0.0040 (6)	−0.0057 (6)
C4	0.0172 (9)	0.0220 (10)	0.0274 (10)	0.0011 (7)	0.0052 (8)	−0.0023 (8)
O5	0.0168 (7)	0.0276 (8)	0.0368 (8)	0.0007 (6)	0.0045 (6)	0.0031 (6)
C5	0.0164 (9)	0.0270 (10)	0.0261 (10)	−0.0009 (8)	0.0037 (8)	−0.0033 (8)
O6	0.0190 (7)	0.0306 (8)	0.0370 (8)	−0.0016 (6)	0.0081 (6)	−0.0033 (7)
C6	0.0229 (10)	0.0363 (12)	0.0266 (10)	−0.0059 (9)	0.0043 (8)	−0.0020 (10)
C7	0.0244 (9)	0.0235 (10)	0.0258 (10)	−0.0023 (8)	−0.0012 (8)	−0.0044 (8)
C8	0.0327 (11)	0.0344 (12)	0.0288 (11)	0.0070 (10)	0.0021 (9)	0.0038 (9)
C9	0.0313 (11)	0.0390 (14)	0.0353 (12)	0.0084 (10)	0.0054 (9)	−0.0012 (10)
O10	0.0399 (9)	0.0585 (11)	0.0354 (9)	0.0012 (9)	0.0143 (7)	−0.0009 (8)
C10	0.0281 (11)	0.0398 (13)	0.0283 (10)	−0.0043 (10)	0.0055 (8)	−0.0046 (10)
C11	0.0376 (13)	0.0344 (12)	0.0335 (12)	0.0000 (10)	0.0054 (10)	0.0080 (10)
C12	0.0326 (11)	0.0263 (11)	0.0355 (12)	0.0058 (9)	0.0033 (9)	0.0040 (10)
C13	0.0294 (11)	0.0350 (13)	0.0346 (11)	0.0058 (10)	0.0044 (9)	−0.0031 (10)
C14	0.0331 (12)	0.0692 (19)	0.0416 (14)	0.0003 (13)	0.0115 (10)	−0.0126 (13)

### Geometric parameters (Å, º)

**Table:**

N1—C7	1.403 (3)	O6—H6O	0.82 (3)
N1—C1	1.444 (3)	C6—H6A	0.9900
N1—C13	1.455 (3)	C6—H6B	0.9900
C1—C2	1.525 (3)	C7—C8	1.395 (3)
C1—H1A	0.9900	C7—C12	1.399 (3)
C1—H1B	0.9900	C8—C9	1.392 (3)
O2—C2	1.209 (3)	C8—H8	0.9500
C2—C3	1.529 (3)	C9—C10	1.380 (3)
O3—C3	1.418 (2)	C9—H9	0.9500
O3—H3O	0.87 (3)	O10—C10	1.382 (3)
C3—C4	1.538 (3)	O10—C14	1.428 (3)
C3—H3	1.0000	C10—C11	1.381 (3)
O4—C4	1.435 (2)	C11—C12	1.383 (3)
O4—H4O	0.80 (3)	C11—H11	0.9500
C4—C5	1.530 (3)	C12—H12	0.9500
C4—H4	1.0000	C13—H13A	0.9800
O5—C5	1.430 (2)	C13—H13B	0.9800
O5—H5O	0.86 (3)	C13—H13C	0.9800
C5—C6	1.518 (3)	C14—H14A	0.9800
C5—H5	1.0000	C14—H14B	0.9800
O6—C6	1.432 (3)	C14—H14C	0.9800
			
C7—N1—C1	119.35 (16)	C5—C6—H6A	108.9
C7—N1—C13	118.38 (17)	O6—C6—H6B	108.9
C1—N1—C13	115.40 (17)	C5—C6—H6B	108.9
N1—C1—C2	114.64 (18)	H6A—C6—H6B	107.7
N1—C1—H1A	108.6	C8—C7—C12	117.14 (18)
C2—C1—H1A	108.6	C8—C7—N1	122.18 (19)
N1—C1—H1B	108.6	C12—C7—N1	120.53 (19)
C2—C1—H1B	108.6	C9—C8—C7	121.5 (2)
H1A—C1—H1B	107.6	C9—C8—H8	119.3
O2—C2—C1	122.7 (2)	C7—C8—H8	119.3
O2—C2—C3	121.04 (19)	C10—C9—C8	120.3 (2)
C1—C2—C3	116.29 (17)	C10—C9—H9	119.9
C3—O3—H3O	109.2 (18)	C8—C9—H9	119.9
O3—C3—C2	110.97 (16)	C10—O10—C14	116.9 (2)
O3—C3—C4	111.77 (16)	C9—C10—C11	118.96 (19)
C2—C3—C4	109.93 (15)	C9—C10—O10	124.8 (2)
O3—C3—H3	108.0	C11—C10—O10	116.2 (2)
C2—C3—H3	108.0	C10—C11—C12	121.0 (2)
C4—C3—H3	108.0	C10—C11—H11	119.5
C4—O4—H4O	108 (2)	C12—C11—H11	119.5
O4—C4—C5	108.19 (15)	C11—C12—C7	121.1 (2)
O4—C4—C3	108.74 (16)	C11—C12—H12	119.4
C5—C4—C3	111.30 (15)	C7—C12—H12	119.4
O4—C4—H4	109.5	N1—C13—H13A	109.5
C5—C4—H4	109.5	N1—C13—H13B	109.5
C3—C4—H4	109.5	H13A—C13—H13B	109.5
C5—O5—H5O	111 (2)	N1—C13—H13C	109.5
O5—C5—C6	108.62 (17)	H13A—C13—H13C	109.5
O5—C5—C4	108.92 (15)	H13B—C13—H13C	109.5
C6—C5—C4	112.72 (16)	O10—C14—H14A	109.5
O5—C5—H5	108.8	O10—C14—H14B	109.5
C6—C5—H5	108.8	H14A—C14—H14B	109.5
C4—C5—H5	108.8	O10—C14—H14C	109.5
C6—O6—H6O	110 (2)	H14A—C14—H14C	109.5
O6—C6—C5	113.26 (17)	H14B—C14—H14C	109.5
O6—C6—H6A	108.9		
			
C7—N1—C1—C2	75.2 (2)	C4—C5—C6—O6	55.7 (2)
C13—N1—C1—C2	−75.3 (2)	C1—N1—C7—C8	24.6 (3)
N1—C1—C2—O2	11.7 (3)	C13—N1—C7—C8	174.25 (19)
N1—C1—C2—C3	−169.53 (16)	C1—N1—C7—C12	−160.0 (2)
O2—C2—C3—O3	−7.0 (3)	C13—N1—C7—C12	−10.3 (3)
C1—C2—C3—O3	174.20 (16)	C12—C7—C8—C9	−1.6 (3)
O2—C2—C3—C4	117.2 (2)	N1—C7—C8—C9	174.0 (2)
C1—C2—C3—C4	−61.6 (2)	C7—C8—C9—C10	0.5 (4)
O3—C3—C4—O4	70.7 (2)	C8—C9—C10—C11	0.8 (4)
C2—C3—C4—O4	−53.0 (2)	C8—C9—C10—O10	−179.7 (2)
O3—C3—C4—C5	−48.3 (2)	C14—O10—C10—C9	3.2 (3)
C2—C3—C4—C5	−172.04 (16)	C14—O10—C10—C11	−177.2 (2)
O4—C4—C5—O5	180.00 (15)	C9—C10—C11—C12	−1.0 (4)
C3—C4—C5—O5	−60.6 (2)	O10—C10—C11—C12	179.5 (2)
O4—C4—C5—C6	59.4 (2)	C10—C11—C12—C7	−0.1 (4)
C3—C4—C5—C6	178.77 (18)	C8—C7—C12—C11	1.4 (3)
O5—C5—C6—O6	−65.1 (2)	N1—C7—C12—C11	−174.2 (2)

### Hydrogen-bond geometry (Å, º)

**Table:**

*D*—H···*A*	*D*—H	H···*A*	*D*···*A*	*D*—H···*A*
O3—H3*O*···O4	0.87 (3)	2.67 (3)	2.962 (2)	101 (3)
O3—H3*O*···O5^i^	0.87 (3)	1.94 (3)	2.747 (2)	153 (2)
O4—H4*O*···O6^ii^	0.80 (3)	1.90 (3)	2.700 (2)	176 (3)
O5—H5*O*···O3^iii^	0.86 (3)	1.86 (3)	2.702 (2)	165 (3)
O6—H6*O*···O5	0.82 (3)	2.56 (3)	2.918 (2)	108 (3)
O6—H6*O*···O4^iv^	0.82 (3)	1.95 (3)	2.704 (2)	154 (3)

Symmetry codes: (i) *x*, *y*+1, *z*; (ii) −*x*, *y*+1/2, −*z*+2; (iii) −*x*+1, *y*−1/2, −*z*+2; (iv) *x*, *y*−1, *z*.

### Supplementary Table S1. Chemical shifts (ppm) of peaks for selected carbon atoms in a ^13^C NMR spectrum of (I) in D_2_O/pyridine (1:1) at 293 K and in the solid state

**Table:**

Carbon	α-pyranose	β-pyranose	α-furanose	β-furanose	acyclic *keto*	solid state
C1	60.97	63.21	61.28	61.28	62.41	57.23
C2	101.13	101.56	108.26	105.62	215.08	210.04
C3	73.62	71.99	84.62	79.73	78.93	77.80
C4	74.92	72.98	80.26	77.42	74.96	69.59
C5	66.66	71.99	85.64	83.77	73.45	69.59
C6	63.7	65.90	64.37	65.14	65.81	64.70
C13	n.r.	42.62	n.r.	42.09	41.85	39.56
